# Anterior Plagiocephaly in an Atypical Case of Apert Syndrome

**Published:** 2013-06

**Authors:** Madhumita Gupta, Ashwin Alke Pai, Abhimanyu Bhattacharya, Ravi Ramachandra, Raghavendra Sawarappa, Subhakanta Mohapatra, Aditya Kanoi

**Affiliations:** Department of Plastic and Reconstructive Surgery, IPGME & R, Kolkata, India

**Keywords:** Apert syndrome, Anterior plagiocephaly, unilateral coronal synostosis, Symmetric syndactyly

## Abstract

Apert syndrome is a congenital craniosynostosis syndrome comprising of bilateral coronal synostosis , symmetric syndactyly of hands and feet and midface hypoplasia. We present an atypical phenotype of this syndrome with right sided unilateral coronal synostosis. However, type I apert hand and other clinical and radiological features suggestthe diagnosis. Genetic analysis revealed an absence of the specific missense mutations in the FGFR 2 gene that is found in patients with this syndrome. We conclude that this patient represented a rare atypical variant of Apert syndrome. Further analysis is required to map the associated genotype.

## INTRODUCTION

Apert syndrome (acrocephalosyndactyly type I) is one of the congenital craniosynostosis syndromes. It was described by Eugene Apert in 1906 who reported a series of 9 cases comprising of a clinical triad of craniosynostosis, symmetric syndactyly of hands and feet and maxillary hypoplasia.^1^ Craniosynostosis is due to the premature fusion of one or more cranial sutures leading to a reduced or asymmetric growth of the cranial vault or base. Virchow in 1851 described that the cessation of growth occurred in a direction perpendicular to the affected sutures.^2^ The shape of the skull in Apert syndrome is variable. However, the most common presentation is the turribrachycephalic skull with a reduced antero-posterior diameter.^3^ We present a case bearing all the typical clinical features of Apert syndrome, but with an anterior plagiocephaly due to unilateral coronal synostosis.

## CASE REPORT

A 7-month old female child presented with the parental complaints of abnormal shape of the head and fusion of the digits of all four limbs since birth. She was the first born child to a normal healthy mother of non-consanguineous marriage. Her father’s age was 39 years. There was no family history of similar complaints or any other congenital anomalies. The child was born by normal vaginal delivery at term after an uneventful antenatal period with no obvious exposure to any mutagen. Her developmental milestones were normal.

**Fig. 1:**
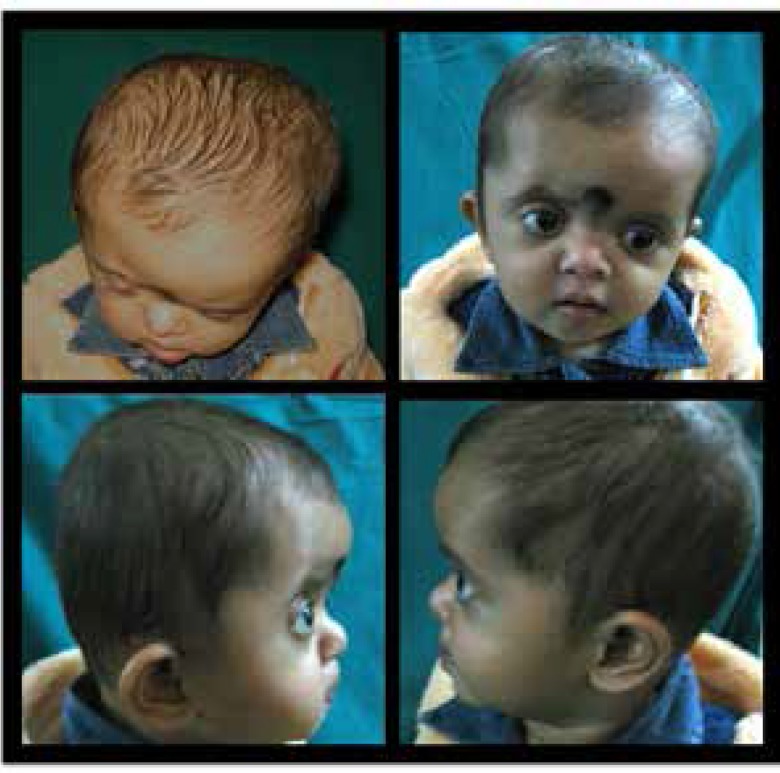
Clinical photographs of craniofacial region: Upper left-top view, upper right-frontal view, lower left-right profile, lower right-left profile

Physical examination of craniofacial region (Figure 1) revealed presence of right sided anterior plagiocephaly with flattened forehead and supraorbital ridge on the ipsilateral side with left sided frontal bossing and bitemporal widening. The ear on the right side was anteriorly displaced. The right eye was exophthalmic with downslanting of the palpebral fissure. The nasal tip was downturned and deviated to the left with depressed bridge of the nose. The lips were cross bow shaped, the palate high-arched and both lower central incisors erupted.

**Fig. 2:**
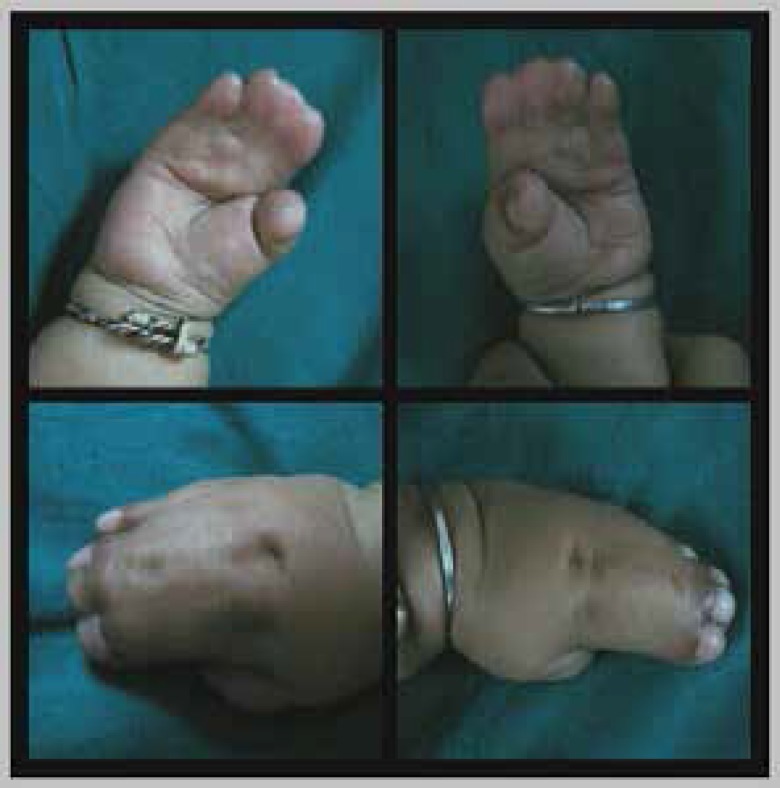
Clinical photographs of hands: Upper left-right hand palmar, upper right-left hand palmar, lower left-right hand dorsal, lower right-left hand dorsal

Both upper limbs showed (Figure 2) symmetric complete simple syndactyly of the 2nd, 3rd and 4th digits and incomplete syndactyly of the 5th digit. The digital plane was flat, thumbs free with radial clinodactyly. The distal joints of the 5th digits were mobile (Type I Apert hand). The feet showed symmetric simple syndactyly of all the toes. Based on these clinical findings a provisional diagnosis of Apert syndrome with atypical cranial contour was made.

**Fig. 3:**
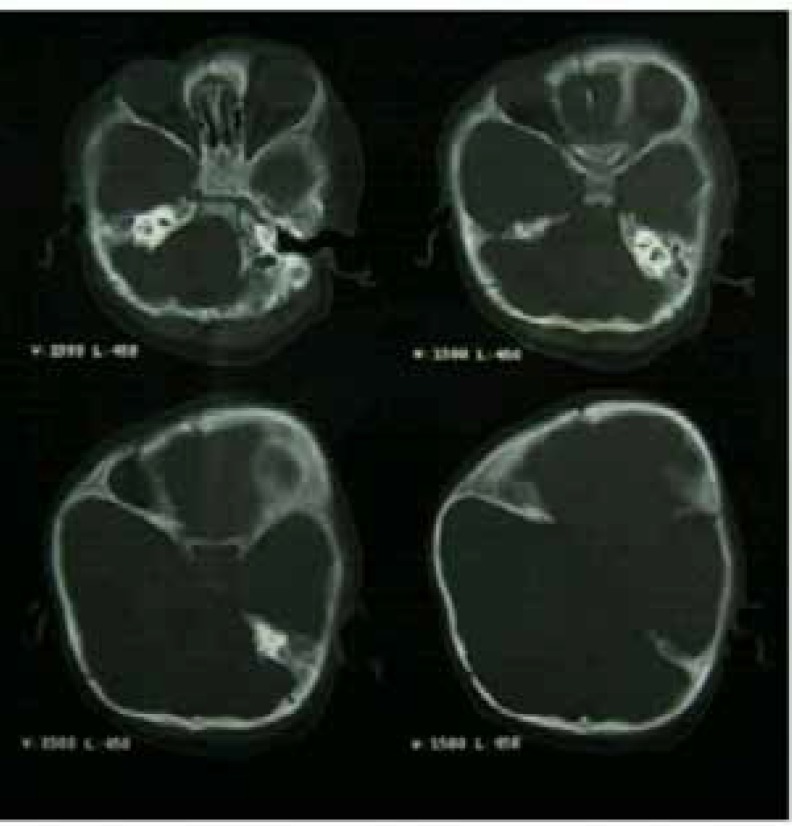
CT scan of cranium and orbit

**Fig. 4:**
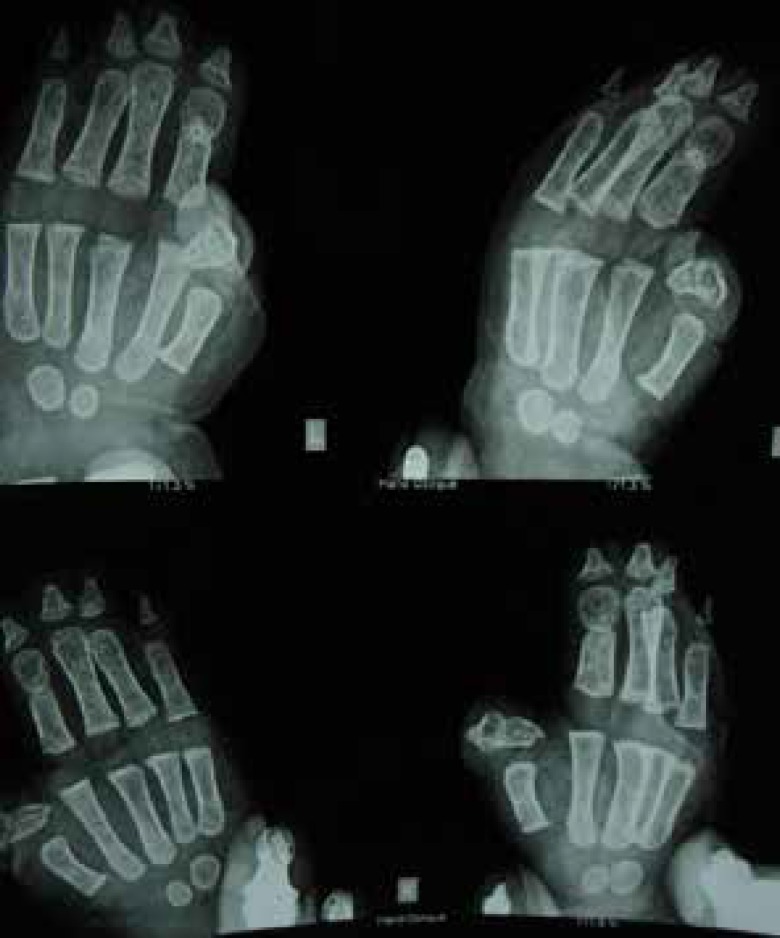
Digital x-ray of both hands (AP and oblique views).

Digital straight X-ray and CT scan of the craniofacial skeleton with 3D reconstruction (Figure 3) showed obliteration of right sided hemicoronal suture with radiologic confirmation of frontal plagiocephaly by cephalometric analysis. Right eye was found to be proptotic with shallow harlequin orbit due to elevated ipsilateral sphenoidal ridge. The ventricles were mildly dilated. Digital X-ray of hands (Figure 4) and feet corroborated the clinical findings of syndactyly with symphalangism. The thumbs showed presence of radially deviated delta phalanx.

X-ray of cervical spine, echocardiography and USG abdomen were all within normal limits.

Genetic analysis of the child failed to detect any mutation involving the FGFR 2 gene.

Bilateral upper limb syndactyly was surgically treated at 7 months of age with release of the 2^nd^ and 4^th^ web spaces. Multistaged surgical correction of the plagiocephalic skull with total fronto-orbital reshaping by multiple incomplete osteotomies, advancements and rigid fixation has been planned at the age of one year. Release of the 3^rd^ web spaces will be addressed at that time.

## DISCUSSION

Apertsyndrome is one of the various congenital craniosynostosis syndromes which may be inherited as an autosomal dominant trait. But mostly it is sporadic with an overall incidence of 50,000 live births. It constitutes about 4.5% of all craniosynostosis cases^4^ and results from new mutations with a paternal age effect.^5^ It typically presents with turribrachycephaly, orbital hypertelorism, exophthalmos and downslanting palpebral fissures. There may be agenesis of corpus callosum, hippocampal abnormalities and progressive hydrocephalus.^6^ Though mental retardation is a common feature many patients present with a normal intelligence quotient.^7^ The maxillae are hypoplastic and retropositioned with high arched palate or bifid uvula. There may be delayed dental eruption, impacted dentition, ectopic or supernumerary teeth, malocclusion or thick gingiva. There is downturned nasal tip, depressed nasal bridge and deviated nasal septum. The lips are trapezoidal in shape and crossbowed . Limbs show symmetric syndactyly, synonychia, symphalangism, brittle discoloured nails with fungal infection. Other skeletal anomalies may include fused upper cervical vertebrae, immobile gleno-humeral or elbow joints and multiple epiphyseal dysplasias.^8^

The typical turribrachycephalic skull in Apert syndrome is due to premature fusion of coronal suture bilaterally with a wide calvarial midline defect from glabella to posterior fontanelle. The antero -posterior diameter is reduced with flat elongated forehead and bitemporal widening and occipital flattening.^3^ However, the skull in this case showed right sided anterior plagiocephalic contour, which is a very rare presentation in an otherwise typical case of Apert syndrome. Only one report from Japan of such a case could be found in the published literature till date.^9^ Anterior plagiocephaly can be deformational or synostotic. The latter may be due to unilateral coronal synostosis (UCS) or rarely frontozygomatic or frontosphenoidal synostosis. UCS is as such uncommon with an overall incidence of 1 in 10,000 live births.^10^ Most of these are sporadic. Rare syndromic cases have been reported to be associated with Seatre-Chotzen syndrome.^11^

The genotype of patients with Apertsyndrome shows remarkably consistent missense substitution mutations in two adjacent amino acids (ser252trp/ser252phe/pro253arg) in the linking region between 2^nd^ and 3^rd^ extracellular Ig domains of FGFR 2 gene in chromosome 10q25-q26.^12^ However, these specific mutations were not detected in this case. The previous reported case from Japan also failed to demonstrate these mutations. It can be concluded that this case represent an atypical variant of Apert syndrome with anterior plagiocephaly and further investigations are needed to determine the associated genotype.

## CONFLICT OF INTEREST

The authors declare no conflict of interest.

## Footenote

Please cite this paper as:

Gupta M, Pai AA, Bhattacharya A, Ramachandra R, Sawarappa R, Mohapatra S, Kanoi A. Anterior Plagiocephaly in an Atypical Case of Apert Syndrome. World J Plast Surg 2013;2(2): 115-118.
